# Woody plant species richness and productivity relationship in a subtropical forest: The predominant role of common species

**DOI:** 10.1371/journal.pone.0306174

**Published:** 2024-07-05

**Authors:** Yudan Sun, Silin Chen, Haofeng Ouyang, Shuang Liu

**Affiliations:** College of Life Science and Technology, LingNan Normal University, Zhanjiang, China; Institute for Biological Research, University of Belgrade, SERBIA

## Abstract

A long-standing key issue for examining the relationships between biodiversity and ecosystem functioning (BEF), such as forest productivity, is whether ecosystem functions are influenced by the total number of species or the properties of a few key species. Compared with controlled ecosystem experiments, the BEF relationships in secondary forest remain unclear, as do the effects of common species richness and rare species richness on the variation in ecosystem functions. To address this issue, we conducted field surveys at five sampling sites (1 ha each) with subtropical secondary evergreen broad-leaved forest vegetation. We found (1) a positive correlation between species richness and standing aboveground biomass (AGB); (2) that common species were primarily responsible for the distribution patterns of species abundance and dominance; although they accounted for approximately 25% of the total species richness on average, they represented 86–91% of species abundance and 88–97% of species dominance; and (3) that common species richness could explain much more of the variation in AGB than total species richness (common species plus rare species) at both the site and plot scales. Because rare species and common species were not equivalent in their ability to predict productivity in the biodiversity-ecosystem productivity model, redundant information should be eliminated to obtain more accurate results. Our study suggested that woody plant species richness and productivity relationship in subtropical forest ecosystem can be explained and predicted by a few common species.

## Introduction

During the past 20 years, an increasing number of studies have investigated how biodiversity influences ecosystem functions and, in turn, affects human society [1–5]. However, to date, no consistent conclusions regarding the nature, shape, and explanatory power of the relationships between biodiversity and ecosystem functioning (BEF) have been reached [6, 7]. The sampling effect theory suggests that some dominant species in communities will affect the BEF relationship [8, 9], while other studies have suggested that the total number of species is much more important than the properties of a few key species [10]. The productivity-diversity theory states that an increase in biomass will lead to the competitive exclusion of subdominant species and a reduction in biodiversity [11]. The biodiversity-ecosystem functioning theory suggests that an increase in species richness will lead to an increase in biomass due to niche complementarity and positive interspecific interactions [12, 13]. However, a recent study suggested that the community assembly affects BEF relationships, although the random sampling effect did not explain those relationships well [6]. Different response patterns of BEF relationships, such as linear, U-shaped, and unimodal, have been described in previous studies [6, 10, 14–17]. Although these theories and discussions have not resulted in definite conclusions, they have increased the awareness of BEF relationships. However, most of these experiments were conducted in small-scale or controlled environments, and such uniform community experiments with perfect constructions and simulations may overestimate the BEF relationship. Secondary forest may yield different results that might be more representative and better reflect the environmental factors affecting plant communities [18, 19].

The composition of the community structure and proportions of species in secondary forest differ from those in controlled environments, therefore the results of BEF relationships differ from natural and controlled experiments. Aboveground biomass (AGB) depends substantially on the dominant or super-dominant species, which are the most abundant and occupy the highest level in the dominance hierarchy [6, 20, 21]. Because rare species are sensitive to changing environmental conditions, they cannot exist for very long in communities, and the suitable habitats for rare species are always rare [22, 23]. Therefore, species richness-productivity patterns are usually driven by the common species. In studies of BEF in secondary forest rare species may be less predictable than common species [23, 24]. Thus, if we do not differentiate the effects of common species from those of rare species, then redundant variance may be introduced when explaining species diversity-productivity relationships; further, any extraneous or confounding information will interfere with the accuracy of the results.

In view of these issues, we selected five sites (1 ha each) in subtropical forests to explore the relationship between species diversity and productivity in larger-scale secondary forest compared to controlled experiments and aimed to differentiate the explanatory effects of common species from those of rare species on biomass variation(The species studied in this paper include only trees, shrubs and woody vines). According to the niche complementarity theory, the existence of different species increases the efficiency of resource utilization, especially in subtropical forests with distinct layers. Therefore, we propose the following hypotheses: (1) There is a significant positive correlation between species richness and aboveground biomass. Additionally, unlike in controlled experiments, the species distribution is uneven and the density of each species is neither the same nor in a fixed proportion in secondary forest; for this reason, the effect of each species on ecosystem functioning is not equivalent. (2) Thus, the richness of common species explains more of the variation in AGB than the richness of all species. In this study, we analyzed the correlation between species richness with aboveground biomass in the subtropical forests, and further explored the role of common species in natural ecosystems on community productivity. This paper also reveals the interpretation and prediction of the relationship between species richness and productivity by a few common species in subtropical forest ecosystems. We proved that AGB is closely related to common species, which provides a new idea for forest management and conservation: future biomass surveys will only measuring the common species, saving a lot of time and cost.

## Materials and methods

### Study area

This study was conducted in the Yinping Mountain Nature Reserve of Guangdong Province (114°14′ E, 22°54′ N). The nature reserve covers an area of 2518.3 ha. The mean annual temperature in this area is 21–22°C. The average minimum temperature of the year is 13–14°C in January, while the average maximum annual temperature is 27–28°C in July. The annual rainfall is 1767.8 mm and occurs primarily from April to September, and the average relative humidity is 79% [25, 26]. In the nature reserve, lateritic red soil occurs below an altitude of 600 m, whereas yellow soil is present at higher elevations [25, 26]. The nature reserve belongs to subtropical monsoon climate, and its vegetation types are diverse, including evergreen broad-leaved forest, lowland evergreen monsoon forest and other types. There are abundant animal and plant resources in the reserve. At present, more than 1500 kinds of wild vascular plants and 170 kinds of terrestrial vertebrates have been found [25, 26]. The forest origins of the five stands in the study area were the artificial forest. In 1999, the harvesting of trees was stopped. In order to promote the forest regeneration, artificial tree species such as *Schima superba* Gardn. et Champ., *Acacia magium* Wild. and *Albizia falcata* (L.) Baker et Merr. were replanted. After planting, close hillsides to facilitate afforestation without human interference, and succession was carried out in a completely natural environment.

### Experimental design and sampling

In 2020, we designated five forest stands at five sites (1 ha each) located more than 2 km from one another in the Yinping Mountain Nature Reserve of Guangdong Province. The sites were coded as M, G, P, X, and J using the initials of their location names, i.e., Mazhukeng, Guangyin Zuolian, Pubuxia, Xiegang, and Jieshi (Table 1). We set up plots on each site using the contiguous grid quadrat method and conducted a survey of trees in each 10×10-m plot. The woody plants we investigated included trees, shrubs and woody vines. We recorded woody plants with a diameter at breast height (DBH) ≥ 1 cm, and measured the DBH, height, and density of all individuals in each plot.

**Table 1:** Plant community and habitat overview.

Community	Mazhukeng	Guangyin Zuolian	Pubuxia	Xiegang	Jieshi
Elevation (m)	280	320	126	333	575
Slope gradient (°)	26	7	32	21	19
Slope aspect	south	-	north by east30°	north	north
Soil forming rock	granite	granite	granite	granite	granite
Forest type	evergreen broad-leaf forest	evergreen broad-leaf forest	evergreen broad-leaf forest	evergreen broad-leaf forest	evergreen broad-leaf forest

### Species abundance classification

For each species (DBH ≥ 1 cm), we calculated the relative abundance (RA), relative frequency (RF), relative dominance (RD), and importance value (IV, IV = RA+ RF+RD) based on the abundance, frequency, and tree basal area. We classified each species as rare or common based on the average relative abundance of <1% (rare) or ≥1% (common) at each site [27, 28].

### Stand volume

We calculated the stand volume using bivariate volume equations provided by the Guangdong Institute of Forest Inventory and Planning [29], as follows:

The bivariate standing tree volume equation for *Pinus massoniana*:

$$V=0.0000798524\times D^{1.74220}\times H^{1.01198}$$

The bivariate standing tree volume equation for *Cunninghamia lanceolata*:

$$V=0.0000697483\times D^{1.81583}\times H^{0.99610}$$

The bivariate standing tree volume equation for *Castanopsis fissa*:

$$V=0.0000629692\times D^{1.81296}\times H^{1.01545}$$

The bivariate volume equations for hardwood species in general:

$$V=0.0000601228\times D^{1.87550}\times H^{0.98496}$$

Where *V* is individual volume (m^3^), *D* is the trunk diameter (cm) measured 1.3 m above the ground, and *H* is the height of the tree (m). The sum of the individual volumes from each site or each plot was used to obtain the stand volume at the site or plot level.

### Biomass calculation

We used the continuous function method of the biomass expansion factor [30] to calculate the AGB: γ=a×X+bWhere *Y* is the biomass of the forest stand (t·ha^-1^), *X* is the stand volume density (m^3^·ha^-1^), and *a* and *b* are constants. The parameters, hence, the *a* and *b* constants, from studies in areas with similar climates were preferably used in the regression equations to calculate the biomass from the volume. The biomass of bamboo was calculated as 22.5 kg·plant^-1^ [31], while the biomass of other forest types was calculated as follows:

The equation for evergreen broad-leaved forest (Gand J Community) in general [32]:

γ=0.7437×X+30.5245

The equation for *Acacia confusa* mixed broad-leaved forest (X Community) [33]:

γ=0.4754×X+30.6034

The equation for hardwood forest (Mand P Community) [30]:

γ=0.7564×X+8.3101

### Data analysis

We calculated species richness, relative abundance (RA), relative frequency (RF), relative dominance (RD), and importance value (IV). Species richness is the number of species at the site or plot level, while importance value is the sum of three species-specific relative values, RA, RF, and RD.

Kruskal-Wallis test for significant difference were used to analyze the variations in species richness for both common species and rare species across sites. We used regression analysis to evaluate the relationship between AGB (the response variable) and common and total species richness (the predictor variables) at both the site and plot scales. All analyses were performed with the software STATISTICA 8.0 (Statsoft. Inc., Tulsa, OK, USA).

## Results

### Species richness and abundance

**Fig 1:**
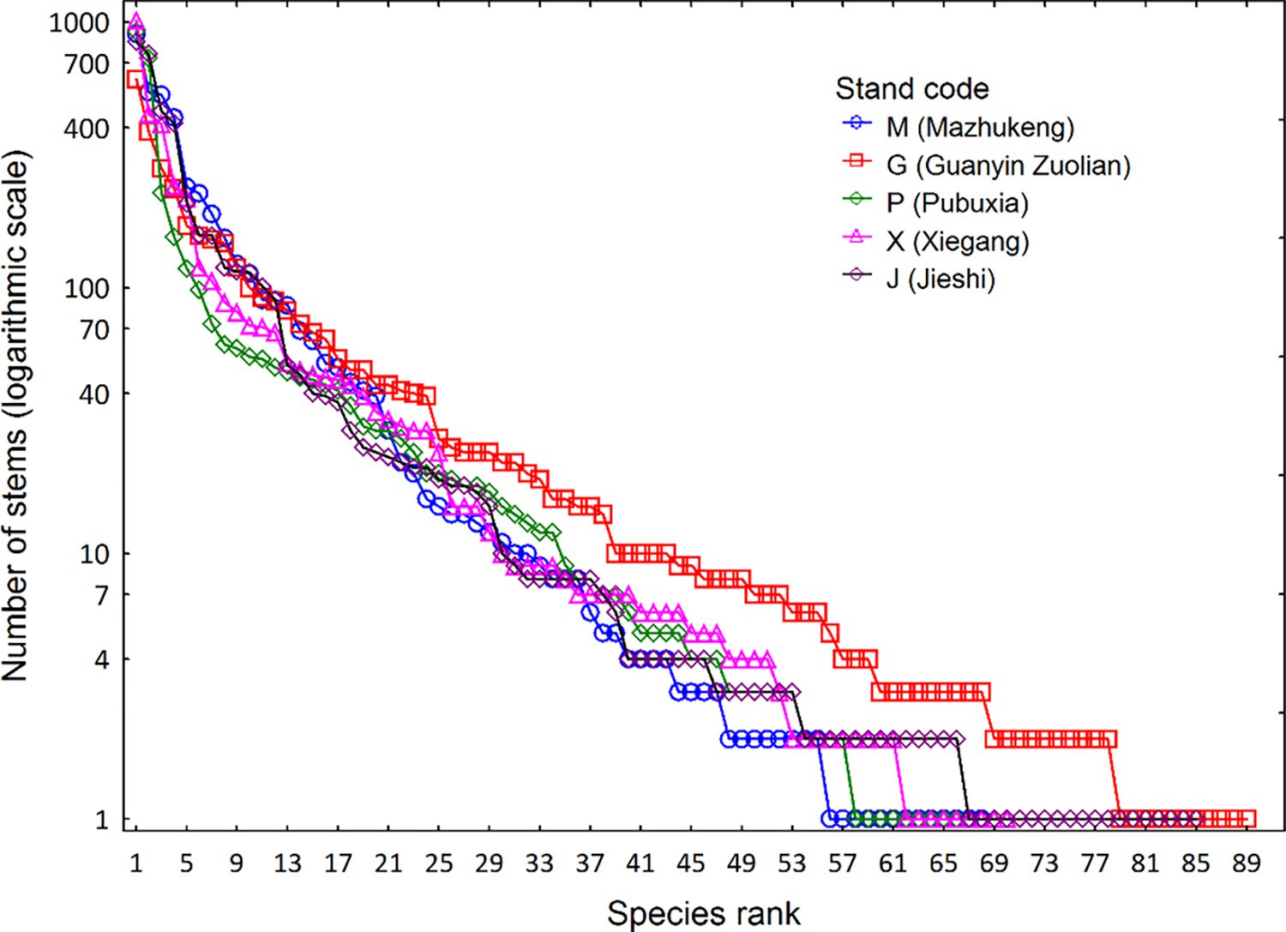
Rank-abundance curves of the five forest stands, showing abundance and dominance patterns.

The rank-abundance curves of the five forest stands followed a log series distribution [34], but the richness, total abundance and abundance of the most dominant species varied (Fig 1). The total number of species for the stands M, G, P, X, and J was 68, 89, 66, 70, and 85; the total abundance for each stand was 4375, 3683, 3289, 3668, and 4177; and number of stems of the most abundant species for each stand is 901, 609, 918, 1012, and 841, respectively (Fig 1). The common species with relative abundances ≥ 1%, by definition, had high plot occurrence, whereas the rare species with lower abundances had low plot frequency. Consequently, the plot average for rare species richness was remarkably lower than for common species richness, although the total rare species richness was markedly greater than the total common species richness at the site scale (Table 2).

**Table 2:** The richness of common species and rare species for each stand and their contributions to relative abundance (RA) and relative dominance (RD) at five sites.

Stand	Common species			Rare species			Total species richness	RA of common species	RD of common species
	Total	Plot average	Plot std. deviation	Total	Plot average	Plot std. deviation			
M^2^	18	8.49 a^1^	2.77	50	2.17 b^1^	1.51	68	91.28	89.50
G	24	8.25 ab	2.46	65	2.71 ab	1.63	89	86.37	88.28
P	18	5.96 c	2.18	48	2.18 b	1.43	66	86.96	93.01
X	19	7.53 ab	1.86	51	2.39 ab	1.44	70	88.82	97.34
J	14	7.28 b	2.00	71	3.06 a	1.82	85	87.02	89.05

^1^Different lowercase letters within a column indicate significant differences (P < 0.05). ^2^M, G, P, X, and J respectively represent Mazhukeng, Guangyin Zuolian, Pubuxia, Xiegang, and Jieshi.

### Community structure

**Fig 2:**
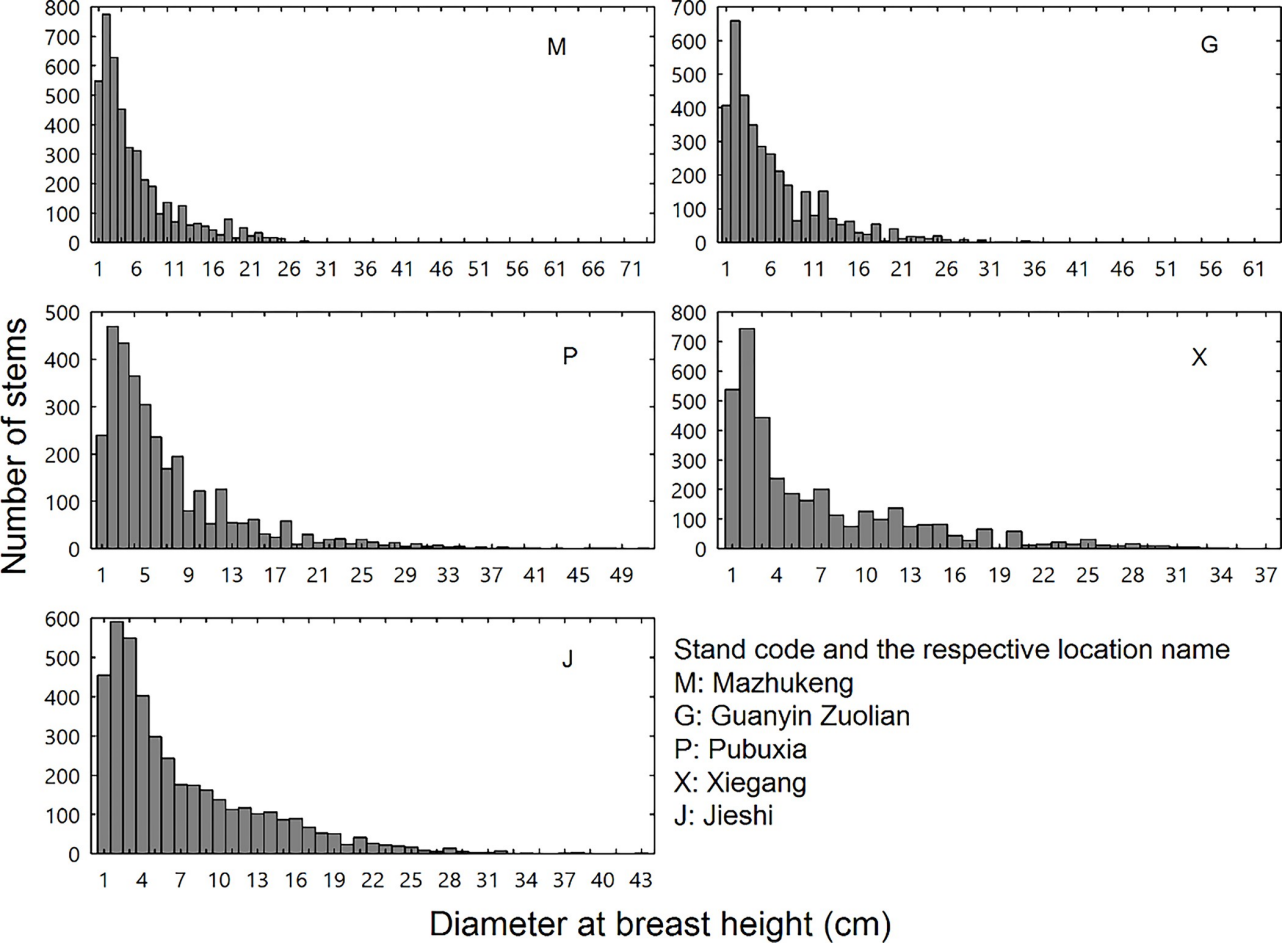
Histograms showing the distribution of diameter at breast height in the five forest stands.

The patterns of tree size distribution varied across the five forest stands (Fig 2). The species with the largest tree DBH for the forest stands M, G, P, X and J were *Schima superba* (73 cm DBH), *Sterculia lanceolata* (63 cm DBH), *Castanopsis fissa* (51 cm DBH), *Castanopsis fissa* (37 cm DBH) and *Machilus chinensis* (37 cm DBH), respectively. For all forest stands, most tree stems were small-sized (Fig 2), while in subtropical forests, the small proportion of large trees contributes heavily to the aboveground biomass [35]. The number of trees with a DBH ≥ 5 cm for the forest stands M, G, P, X and J was 1966, 1832, 1782, 1705, and 2179, respectively (Fig 2).

The most abundant and frequent species differed across the five stands based on the abundance and frequency ranks. However, when ranked by the overall estimate of importance, *Schima superba* had the highest value and thus is the most dominant species in the stands M, G, P, and J, while the dominant species for stand X was *Acacia mangium* (Table 3). Subdominant species varied across the five stands (Table 3). By representing a small proportion of the total species richness, these dominant and subdominant species accounted for a large proportion of the importance value (Table 3). Similarly, the small proportion of common species (approximately 25% of the total species in each stand) was responsible for the overwhelming proportion of abundance and dominance (Table 2).

**Table 3:** Structural parameters of dominant species in each of the five stands.

Stand	Species	RA^1^	RF^1^	RD^1^	IV^3^
M^2^	*Schima superba* Gardn. et Champ.	12.20	7.60	40.90	60.69
	*Schefflera heptaphylla* (L.) Frodin	10.00	6.94	15.45	32.39
	*Litsea rotundifolia* var. *oblongifolia* (Nees) Allen	20.62	7.97	3.02	31.61
	*Itea chinensis* Hook. et Arn.	12.17	7.22	5.75	25.14
	*Cinnamomum parthenoxylon* (Jack) Meisn.	2.06	3.75	6.79	12.61
	*Acronychia pedunculata* (L.) Miq.	5.17	3.38	3.85	12.40
	*Psychotria rubra* Poir	5.47	6.29	0.51	12.26
	*Aporusa dioica* (Roxb.) Muell.-Arg.	3.52	5.07	1.64	10.23
G	*Schima superba* Gardn. et Champ.	16.51	5.93	31.11	53.55
	*Machilus chinensis* (Champ. ex Benth.) Hemsl.	7.63	5.66	12.36	25.65
	*Aporusa dioica* (L.) Miq.	10.53	6.66	3.16	20.35
	*Adenanthera pavonina* L.	4.13	4.93	5.63	14.69
	*Psychotria rubra* Poir	6.41	5.93	0.53	12.87
	*Acronychia pedunculata* (L.) Miq.	4.64	4.29	3.35	12.28
	*Cinnamomum parthenoxylon* (Jack) Meisn.	2.23	3.10	6.77	12.10
	*Syzygium rehderianum* Merr. et Perry	3.99	5.57	0.55	10.11
P	*Schima superba* Gardn. et Champ.	22.26	10.95	37.60	70.80
	*Itea chinensis* Hook. et Arn.	27.91	11.07	13.69	52.67
	*Castanopsis fissa* Rehd.et Wils.	6.93	5.66	31.20	43.79
	*Styrax suberifolius* Hook. et Arn.	3.59	3.69	1.59	8.87
	*Litsea rotundifolia* var. *oblongifolia* (Nees) Allen	4.71	3.44	0.65	8.81
	*Aporusa dioica* (L.) Miq.	2.98	4.06	0.70	7.74
	*Sapium discolor* (Champ. ex Benth.) Muell.-Arg.	1.79	3.94	0.97	6.70
	*Schefflera heptaphylla* (L.) Frodin	1.64	3.57	1.26	6.47
X	*Acacia mangium* Willd.	27.59	9.99	57.42	95.00
	*Vernicia montana* Lour.	6.57	8.07	18.71	33.35
	*Litsea cubeba* (Lour.) Pers.	11.26	9.28	2.61	23.16
	*Cunninghamia lanceolata* (Lamb.)Hook.	12.27	5.35	1.18	18.80
	*Castanopsis fissa* Rehd.et Wils.	3.24	4.64	6.91	14.80
	*Evodia lepta* (Spreng.) Merr.	5.92	5.85	0.71	12.48
	*Falcataria moluccana* (Miq.) Barneby Grimes	1.06	2.52	4.08	7.66
	*Schima superba* Gardn. et Champ.	2.89	3.13	1.53	7.55
J	*Schima superba* Gardn. et Champ.	20.13	9.01	28.75	57.90
	*Machilus chinensis* (Champ. ex Benth.) Hemsl.	11.06	8.43	23.80	43.29
	*Itea chinensis* Hook. et Arn.	18.15	8.82	3.80	30.77
	*Rhodoleia championii* Hook. f.	6.80	5.33	15.61	27.74
	*Adina pilulifera* (Lam.) Franch	4.96	6.20	0.80	11.96
	*Diospyros morrisiana* Hance	2.85	5.72	2.15	10.71
	*Myrsine seguinii* H. Lv.	3.78	5.33	1.19	10.30
	*Dendropanax proteus* (Champ.) Benth.	2.73	4.26	1.17	8.16

^1^Abbreviations: RA = relative abundance; RF = relative frequency; RD = relative dominance; IV = importance value.

^2^M, G, P, X, and J respectively represent Mazhukeng, Guangyin Zuolian, Pubuxia, Xiegang, and Jieshi.

^3^ The top 8 important values of each plot were selected as the dominant species.

### Relationship between species richness and AGB

The number of common species in all the stands was much smaller than the number of rare species. The ratio of common species to rare species was approximately 1:3 at the stand level. However, not only was the frequency of occurrence of common species much greater than that of rare species, but the common species also contributed the most to the abundance (the range of RA was 86.37–91.28) and relative dominance (the range of RD was 88.28–97.34) (Table 2). Based on these results, the common species determined the community structure and thus determined the AGB.

**Fig 3:**
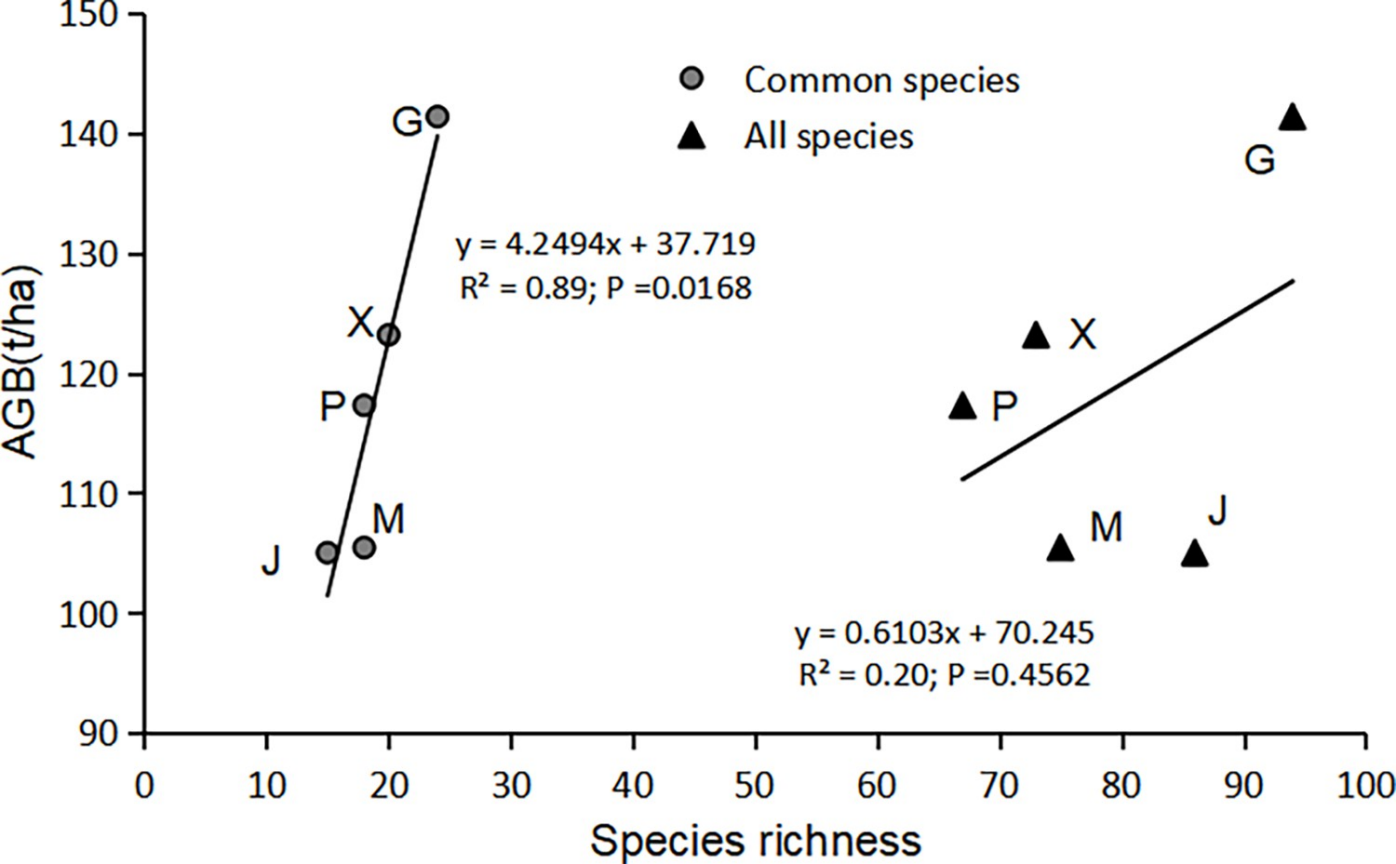
Relationship between species richness and AGB at the site scale. Abbreviation: AGB, Aboveground biomass; M, G, P, X, and J respectively represent Mazhukeng, Guangyin Zuolian, Pubuxia, Xiegang, and Jieshi.

**Fig 4:**
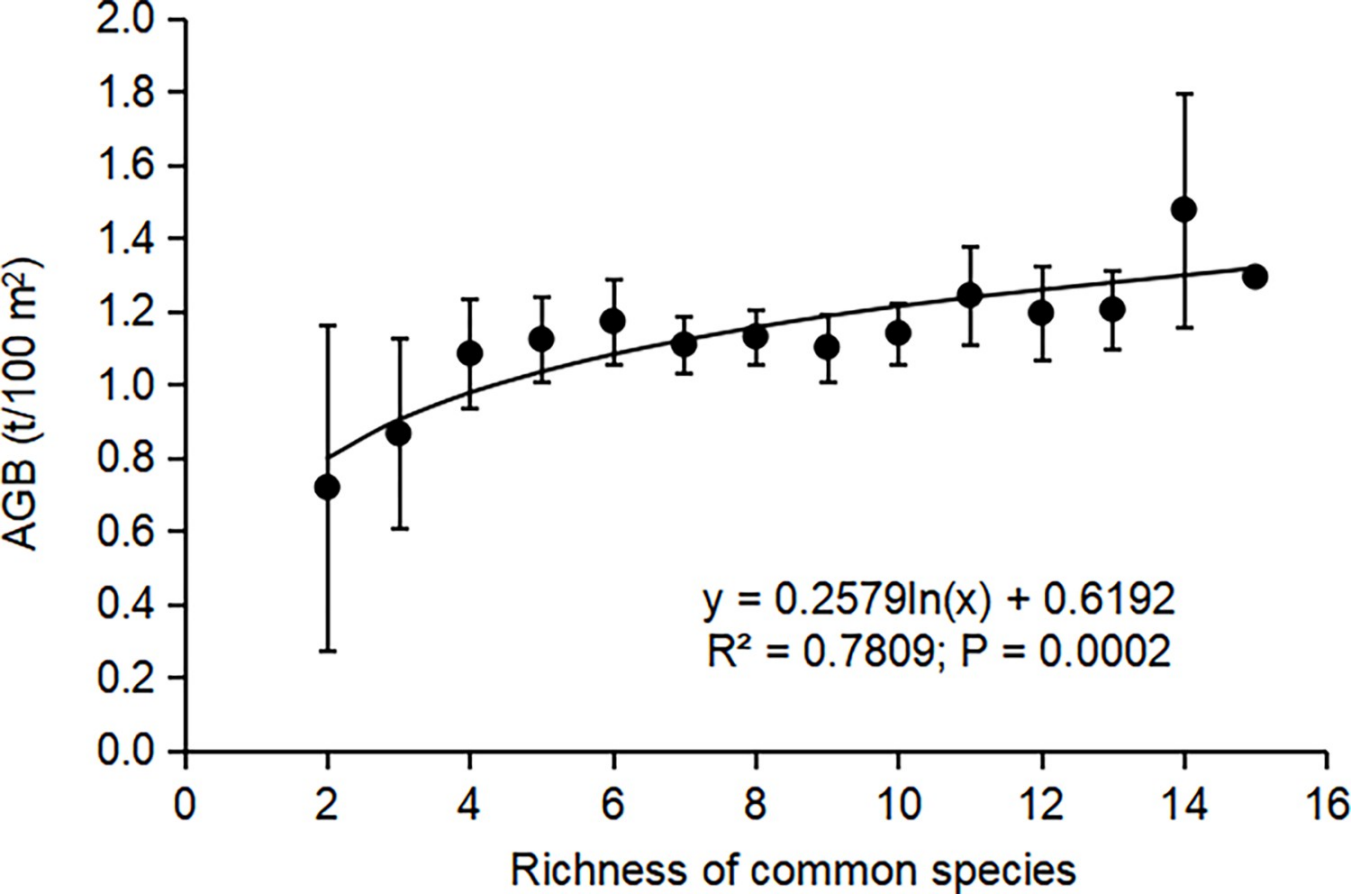
Relationship between the common species richness and AGB at the plot scale. Plotted values are means ± standard error. Abbreviation: AGB = Aboveground biomass.

**Fig 5:**
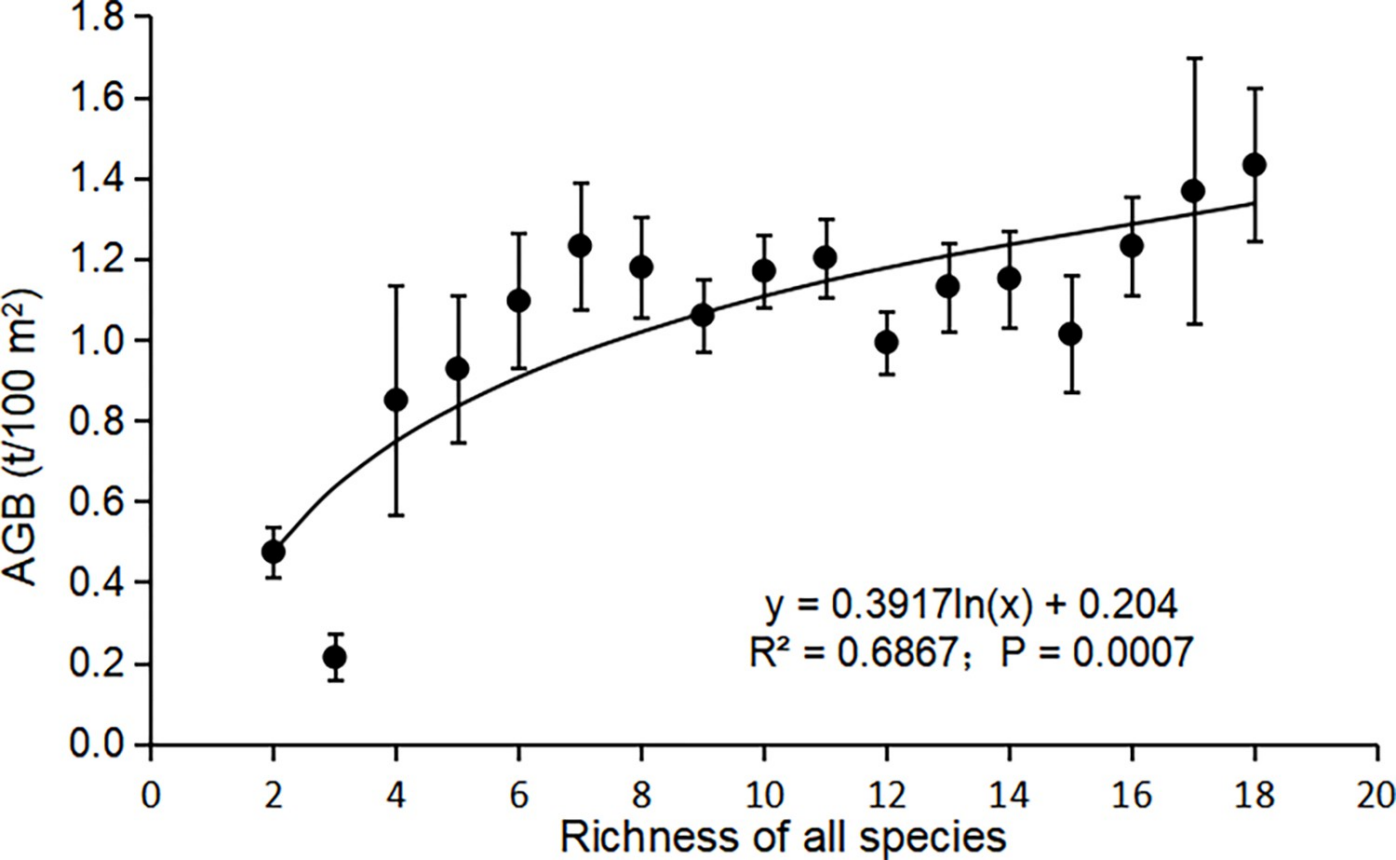
Relationship between the richness of all species and AGB at plot scale. Plotted values are means ± standard error. Abbreviation: AGB = Aboveground biomass.

The Shapiro-Wilk test has been performed on common species richness, total species richness and aboveground biomass in each stand, and the three variables all obey the normal distribution (Table 4). At the site scale, although the correlation between total species richness and AGB was not significant (Fig 3; R^2^ = 0.20, P = 0.4562), there was a significant positive correlation between the common species richness and AGB (Fig 3; R^2^ = 0.89, P = 0.0168). At the plot scale, regression analysis revealed that the common species richness could better explain the variation in AGB (Fig 4; R^2^ = 0.78, P = 0.0002) compared to the total species richness (Fig 5; R^2^ = 0.69, P = 0.0007). Thus, eliminating the effect of rare species yielded more significant positive correlation in our study of the relationship between species richness and AGB in the subtropical forest ecosystems.

**Table 4:** Shapiro-Wilk test of common species, total species and aboveground biomass in each.stand.

Statistical index	Shapiro-Wilk test		
	Statistic	df	Sig.
Common species	.950	5	0.735
All species	.946	5	0.707
AGB (t/ha)	.900	5	0.411

Abbreviation: AGB = Aboveground biomass.

## Conclusions and discussion

### Relationships between species richness and productivity

The relationships between species richness and productivity show differently in different plant community types and under different environmental conditions. An earlier study reported a statistical analysis of approximately 200 experimental results for the BEF relationships and found that 30% were unimodal curves, 26% were positive linear relationships, and 12% were negative linear relationships, whereas the last 32% showed no obvious relationships [36]. However, in the grassland ecosystem, the experimental data tended to support the hypothesis that species richness and productivity are positively correlated [10, 37]; thus, increasing niche separation would lead to complementation or promotion within species. Species diversity also promoted nutrient cycling within the ecosystem, and the high-yield species had a greater probability of survival [38–40]. These mechanisms and theories could be treated as an explanation for the hypothesis that species richness and productivity are positively correlated. This hypothesis was also supported by the results of our study. In our study, species richness was positively correlated with ecosystem productivity at both the site and plot scales, which is consistent with our first hypothesis. This might be due to the environmental conditions in the subtropical areas with ample precipitation and sunshine, in which this study was conducted. Some researchers have suggested that highly productive habitats develop more easily in tropical areas with high temperatures and precipitation [41]. Therefore, the tropical and subtropical areas should have larger species pools and higher productivity and will likely fit better with the positive linear model [11]; whereas the unimodal model is more likely to be well suited to temperate regions.

### The role of common species and rare species in explaining BEF relationships

The results of field experiments differ from those of controlled experiments, as it is not possible to precisely control environmental conditions nor to generate a uniform species composition and distribution in the former [42, 43]. The impact of rare species on ecosystem functions has been included in the biodiversity-ecosystem productivity model used in controlled experiments. In field experiments, when rare species and common species are considered equivalent, this leads to redundant information in the biodiversity-ecosystem productivity model that reduces its accuracy and, as a result, we cannot fully understand the BEF relationships. Moreover, compared with strictly controlled experiments, experiments in secondary forest are limited by unstable environmental conditions, the initial species composition, and limited resources, all of which strongly vary [39, 44]. Furthermore, a sufficiently long succession time could drive competition between species and lead to the coexistence of species groups with different resource utilization efficiencies and varying performance during the long-term natural succession. Our results showed that common species, which accounted for approximately 25% of the species pool in the sample plot, contributed to 86.37–91.28% of total plant abundance and 86.37–97.34% of woody plant basal area, which is referred to as woody plant dominance. Clearly, common species determined the distribution patterns of community abundance and dominance, which directly affected the community structure and properties. At the site scale, the richness of all species was not able to explain the variation in biomass, whereas the common species richness was able to explain 89% of the variations in biomass. At the plot scale, common species richness better explained and predicted the variation in biomass than did total species richness. Therefore, in the biodiversity-ecosystem productivity model for a secondary forest, the rare species richness was in one way redundant because its effect was not equivalent to that of common species richness when explaining variation in productivity. When the influence of rare species is included, the biodiversity-ecosystem productivity model contains redundant information. These results are consistent with our second hypothesis. From this perspective, it is possible that the BEF relationship is more likely to be determined by a few key species.

## Supporting information

S1 Data (XLS)
